# Systemic Antibiotics in the Surgical Treatment of Peri‐Implantitis: Impact on the Salivary Microbiome

**DOI:** 10.1111/jcpe.70143

**Published:** 2026-05-25

**Authors:** Caroline Riben Grundström, Bodil Lund, Nagihan Bostanci, Angelika Silbereisen, Alexandra Pennhag, Ina Schuppe Koistinen, Yinghua Zha, Anastasios Damdimopoulos, Georgios N. Belibasakis, Margareta Hultin

**Affiliations:** ^1^ Department of Periodontology, Specialist Clinic Kaniken Public Dental Health Service Uppsala Sweden; ^2^ Department of Dental Medicine Karolinska Institutet Huddinge Sweden; ^3^ Medical Unit of Plastic Surgery and Oral and Maxillofacial Surgery, Department for Oral and Maxillofacial Surgery and Jaw Orthopedics Karolinska University Hospital Stockholm Sweden; ^4^ Department of Microbiology, Tumor and Cell Biology Karolinska Institutet Solna Sweden; ^5^ Department of Medical Epidemiology and Biostatistics Karolinska Institutet Solna Sweden; ^6^ Bioinformatics and Expression Analysis Core Facility, Department of Medicine Huddinge Karolinska Institutet Huddinge Sweden

**Keywords:** amoxicillin, metronidazole, microbiota, penicillin V, saliva

## Abstract

**Aim:**

To exploratorily compare the shifts in the salivary microbiome composition after administration of two combined systemic antibiotic regimens used in the surgical treatment of peri‐implantitis.

**Materials and Methods:**

A subset of 27 patients treated surgically for peri‐implantitis with an adjunctive 7‐day course of systemic antibiotics were included (group A, amoxicillin and metronidazole; group B, phenoxymethylpenicillin and metronidazole). Unstimulated saliva was collected before surgery followed by 8 days, 14 days, 6 months and 12 months post surgery. Microbiome profiling was performed using standardised and automated pipelines for DNA extraction and whole genome shotgun sequencing (WGS).

**Results:**

WGS identified 498 species across 194 genera and 16 phyla, with Firmicutes and Actinobacteria being the most abundant. A distinct decrease in alpha diversity was observed on Day 8 relative to baseline across both antibiotic regimens, with signs of richness recovery by Day 14. Alpha and beta diversity analyses showed no statistically significant differences between interventions over the 12‐month observation period.

**Conclusion:**

Both adjunctive antibiotic regimens applied in the surgical treatment of peri‐implantitis caused comparable ecological disturbances in the salivary microbiome, with no microbiological or clinical evidence supporting the superiority of one regimen over the other.

**Trial Registration:** ClinicalTrials.gov identifier: NCT02185209

## 1 Introduction

Peri‐implantitis is a biofilm‐associated inflammatory condition affecting the soft and hard tissues surrounding a dental implant. Key features include bleeding on probing (BOP) with or without suppuration on probing (SOP), increased probing depths (PDs) and progressive marginal bone loss, which can ultimately lead to implant failure if not properly treated (Berglundh et al. 2018; Tomasi and Derks 2022).

The presence of dysbiosis within the peri‐implant biofilm is identified as the primary risk indicator for peri‐implant disease, although specific bacterial signatures have not been fully elucidated (Belibasakis and Manoil 2021; Carvalho et al. 2023; Chun Giok and Menon 2023; Schwarz et al. 2018).

Surgical treatment of peri‐implantitis with adjunctive systemic antibiotics may yield superior short‐term clinical and microbiological results; however, they offer limited long‐term benefits (Carcuac et al. 2017; Hallström et al. 2017; Riben Grundström et al. 2024). Additionally, the impact on human microbiome dysbiosis and subsequent development of antibiotic‐resistant genes have led to the non‐recommendation in recent clinical guidelines (Herrera et al. 2023).

Saliva harbours a plethora of diverse microbiota and plays a pivotal role in shaping the oral microbiome and maintaining oral homeostasis. As the second most diverse habitat with over 700 cultured species, its microbiome has been highlighted as a potential reservoir for AMR‐related genes (Paster et al. 2006). While next‐generation sequencing (NGS) of 16S rRNA genes enables genus‐ or species‐level taxonomic profiling, whole genome sequencing (WGS) detects microbial signatures linked to functional and metabolic changes (Quince et al. 2017).

A healthy oral core microbiome comprises five phyla: Actinobacteria, Bacteroidetes, Firmicutes, Fusobacteriota and Proteobacteria (Zaura et al. 2009). The primary genera include *Streptococcus*, *Fusobacterium*, *Haemophilus*, *Neisseria*, *Prevotella* and *Rothia* (Joseph and Curtis 2021). While minor ecological changes can be manageable by the host, prolonged disruptions due to age, diet changes, antibiotics, female hormones or antiseptic mouthwashes may alter the microbiota and lead to periodontitis and caries (Bartsch et al. 2025; Bescos et al. 2020; Bostanci et al. 2021; Mira et al. 2017; Sedghi et al. 2021) as well as systemic conditions such as cardiovascular and neurodegenerative diseases (Fogelholm et al. 2023; Zhong et al. 2024).

Compared to the gut microbiome, the oral microbiome has been the subject of fewer investigations on the long‐term impact of antibiotics (Gomez‐Arango et al. 2017; Jakobsson et al. 2010; Menon et al. 2019; Raju et al. 2020). While the effect of amoxicillin with or without metronidazole (Bizzarro et al. 2016; Hagenfeld et al. 2018; Khalil et al. 2016; Lazarevic et al. 2013; Zaura et al. 2015) has been more frequently studied, studies on the effects of penicillin V (phenoxymethylpenicillin) on the oral microbiota are limited (Heimdahl et al. 1982; Heimdahl et al. 1979; Nord and Heimdahl 1986; Raju et al. 2020). The Swedish National Recommendations for antibiotic treatment of oral infections suggests the use of narrow‐spectrum phenoxymethylpenicillin (penicillin V) alone or in combination with metronidazole for adjunctive local infection control in selected cases. Phenoxymethylpenicillin exhibits antimicrobial efficacy primarily against Gram‐positive bacteria. Compared to amoxicillin, its efficacy against Gram‐negative bacteria is limited, designating it as one of the most narrow‐spectrum penicillin variants available. Consequently, phenoxymethylpenicillin is regarded as having a lower potential to promote antibiotic resistance than broader spectrum agents such as amoxicillin (Bush and Bradford 2016).

To the best of our knowledge, this study is the first to evaluate the effect of amoxicillin and metronidazole or phenoxymethylpenicillin and metronidazole on the saliva microbiome after peri‐implant surgery using whole genome sequencing (WGS) technology.

Thus, this study aimed to comparatively evaluate both short‐ and long‐term changes in the salivary microbiome structure and composition after administration of two combined systemic antibiotic regimens used in surgical treatment of peri‐implantitis.

## 2 Materials and Methods

The main materials and methods used are listed below. For a more detailed description, see Materials and Methods in Supporting Information.

### 2.1 Trial Design

This study was undertaken as a blinded, single‐centred, two‐armed, exploratory investigation of the saliva microbiome with a 12‐month follow‐up at the Department of Periodontology, Public Dental Health Clinic Kaniken, Uppsala, Sweden.

### 2.2 Participants

The study was approved by the Ethical Review Board, Stockholm, Sweden (Dnr 2014‐1331‐31‐1) and the Swedish Medical Products Agency (EudraCT no 2013‐004724‐11) and registered at https://clinicaltrials.gov (NCT02185209, https://clinicaltrials.gov/study/NCT02185209) 9 July 2014. CONSORT guidelines were followed (Hopewell et al. 2025). A total of 84 patients with peri‐implantitis were included for resective peri‐implant surgery with systemic antibiotics or placebo in a three‐armed randomised, placebo‐controlled trial previously published (Riben Grundström et al. 2024). A subset of 27 patients randomised into two antibiotic groups collected saliva samples at five timepoints over the 12‐month period; Group A, amoxicillin (Sandoz) 500 mg and metronidazole (Sanofi) 400 mg, three times daily; Group B, phenoxymethylpenicillin (Meda) 800 mg × 2 and metronidazole (Sanofi) 400 mg, three times daily for 7 days. All participants gave oral and written consent to participate. Inclusion and exclusion criteria were defined as previously published, but we further excluded patients with xerostomia and slow bowel movement (S1.1 in Supporting Information).

### 2.3 Intervention

Following baseline clinical examination and non‐surgical therapy, the surgical procedure was scheduled. Patients received 1 g of paracetamol (AlvedonGlaxoSmithKlein) and rinsed with chlorhexidine for 1 min (FluxPro 0.12% KavoPharma AB, Sweden) pre‐operatively. Upon flap elevation, the implant surfaces were decontaminated by titanium curettes and ultrasound followed by 3% hydrogen peroxide and saline irrigation according to the study protocol. Post‐operative care included abstaining from brushing the surgically treated area and rinsing with 10 mL of 0.12% chlorhexidine twice daily for 14 days. All examinations and surgeries were performed by one periodontist (C.R.G.) blinded to the allocation.

### 2.4 Outcomes

Primary outcome was qualitative and quantitative microbial changes in the saliva over 12 months. Secondary outcome was treatment success (defined as PPD ≤ 5 mm, BOP in ≤ 1 site, absence of SOP and absence of additional bone loss > 0.5) at the 12‐month follow‐up.

### 2.5 Sample Size

No data were available to assess the impact of surgical treatment of peri‐implantitis with systemic antibiotics on the saliva microbiome at the time of study planning; consequently, the subset selection was based on prior descriptive oral microbiome studies (Rashid et al. 2013; Sullivan et al. 2001). Patients were initially randomly assigned by a computer‐generated list divided in blocks of three to a three‐armed study (Riben Grundström et al. 2024). The first set of patients randomised to the two antibiotic arms in that study were included for saliva collection. To address the anticipated higher drop‐out rates, ethically approved compensatory measures permitted further inclusion until each group comprised 12 subjects with completed 12‐month evaluation. Because of prolonged enrolment periods, a total of 27 patients were eventually included. For details of post hoc power calculation, see S1.2 in Supporting Information.

### 2.6 Saliva Collection and Processing

Patients were instructed by the study nurse to self‐collect a minimum of 5 mL un‐stimulated saliva at five timepoints: within a week before surgery (baseline saliva) followed by 8 days, 14 days, 6 months and 12 months after surgery. Samples were taken in the morning before breakfast, tobacco usage and toothbrushing and collected in sterile plastic tubes labelled with initials, randomisation number and date. A study nurse called the patients to remind them about the saliva collection and delivery to the clinic in conjunction with their upcoming clinical follow‐up. The samples were temporarily stored at −20°C in the patient's home and in the dental clinic, and then transported and stored at −80°C in the core facility until further use (S1.3 in Supporting Information).

Samples were defrosted on ice (+4°C), their total volume was noted and were transferred to 1.5‐mL Eppendorf tubes. Samples were centrifugated at 4000 rpm for 20 min at 4°C. The saliva supernatants (SNs) were transferred into two new Eppendorf LoBind tubes as 250‐μL aliquots. The remaining saliva pellet was dissolved in 500 μL or 1 mL PBS with a RNase inhibitor (R7397 4°C j41) at 1:500 dilution and briefly vortexed. The SN and pellet samples were frozen down to −20°C after which they were moved to −80°C for long‐term storage. The pellet samples were transported to the Centre of Translational and Molecular Research (CTMR), Solna, Karolinska Institutet, Sweden, for further DNA extraction and sequencing.

### 2.7 DNA Extraction

Samples were thawed and mixed with 300 μL DNA/RNA shield. Next, 800 μL was transferred to a ZR BashingBead lysis tube (Zymo Research). All samples were bead‐beaten in a FastPrep24 5G machine. After centrifugation, 200 μL of each sample was purified using the Tecan Fluent according to the ZymoBIOMICS 96 MagBead DNA protocol. Elution was carried out with 50 μL of EB buffer (Qiagen), and the extracted DNA was stored at −20°C until analysis (S1.4 in Supporting Information).

### 2.8 Library Preparation

Genomic DNA was prepared using the MGIEasy FS DNA set with 50 ng DNA input, as per the manufacturer's protocol. For saliva samples with low initial microbial DNA, a spike‐in, *Alicyclobacillus acidiphilus*, was added to improve the success rate when preparing libraries. For samples with concentrations meeting the required 50 ng DNA input, 0.5 ng of *A. acidiphilus* was included, which equals 1% of the total DNA input. This species was selected because of its absence in typical human samples.

Library quality was performed with TapeStation D1000 (Agilent, USA) and quantity assessed using QuantIT HighSensitivity dsDNA Assay on a Tecan Spark (Tecan, Switzerland). Equal amounts of DNA were pooled, circularised using the MGI Easy Circularisation kit (MGI Tech, China) and sequenced (150 bp paired‐end) on the DNBSEQ G400 sequencing instrument (MGI Tech, China) (S1.5 in Supporting Information). The sequence data have been submitted to the GenBank (www.ncbi.nlm.nih.gov/genbank/) database (Benson et al. 2018) under accession number PRJEB102458.

### 2.9 Bioinformatic Analysis

Shotgun sequencing data was processed using version 0.7.0 of the StaG‐mwc Snakemake workflow (Boulund et al. 2023). Data was quality‐controlled and filtered using Fastp (Chen 2023). Human reads were removed by matching to the GRCh38 human reference database using Kraken2 (Wood et al. 2019). Samples that did not achieve 100 k non‐human reads were excluded from downstream analyses. Reads were taxonomically classified using MetaPhlAn4 (Blanco‐Míguez et al. 2023), with the MetaPhlAn vOct22 database. Processing results were aggregated in MultiQC version 1.11 (Ewels et al. 2016).

### 2.10 Statistical Analysis

Clinical, demographic and treatment success analyses were conducted using two‐tailed Student's *t*‐tests, *χ* ^2^ test and Fisher's exact test, with statistical significance defined as *p* < 0.05. MetaPhlAn4‐generated abundance profiles were imported into R (v4.4.2)/Bioconductor (v3.21) for preprocessing (Chen et al. 2020; Portik et al. 2022). The genus *Alicyclobacillaceae* was removed and remaining data were re‐normalised with TMM/TMMwsp in edgeR. Species with low/zero counts were excluded using the filterByExpr function.

Microbial richness was analysed with generalised linear mixed models (GLMMs) implemented in glmmTMB package with negative binomial (nbinom2) distribution.

Shannon diversity and evenness were analysed using linear mixed models (LMMs) and ANOVA in R using the lmer and ANOVA functions from the lmerTest package, incorporating time and intervention as fixed effects, with the patientsID as random intercept. This was followed by ANOVA using Satterthwaite's approximation for denominator degrees of freedom.

Beta‐diversity was assessed using principal coordinates analysis (PCoA) performed with vegan (Oksanen et al. 2025), via the wcmdscale function on a Bray–Curtis dissimilarity matrix generated with the vegdist function. Analysis of variance (PERMANOVA) was performed with the adonis2 function (99,999 permutations).

For differential species analysis, relative abundances were converted to estimated counts by multiplying each species' relative abundance by the corresponding sample's total number of non‐host sequencing reads, to account for sequencing depth differences and to facilitate the use of edgeR (Robinson et al. 2010) (S1.6 in Supporting Information). Statistically significant results are reported as FDR‐adjusted (Benjamini‐Hochberg) *q* values < 0.05.

Visualisations were created in R via ggplot2 (Wickham 2016) and the ComplexHeatmap (Gu et al. 2016).

## 3 Results

### 3.1 Clinical Characteristics of Included Patients and Implants

**FIGURE 1:**
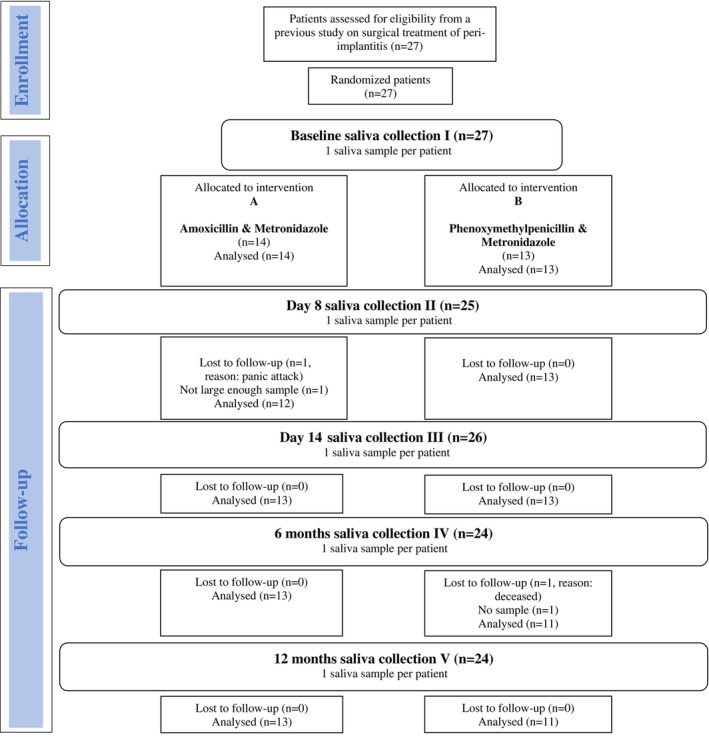
Flowchart.

Table 1 summarises the clinical data of the 27 patients. In Group A, by Day 8, one patient was lost to follow‐up, and another patient provided insufficient saliva due to technical issues but resumed proper sampling. In Group B at 6 months, one patient died and another missed both 6‐and 12‐month samples. By 12 months, a total of 126 samples were collected, with 23 patients providing complete samples across all five timepoints (115 samples) (Figures 1 and S1).

**TABLE 1:** Baseline characteristics of included patients and implants.

Group	All	A	B	*p*
Patients, *n*	27	14	13	
Age (years)	62.3 (18.1)	59.9 (17.6)	64.8 (19.0)	0.499^a^
Gender: M (male), F (female), *n*	M 10, F 17	M 5, F 9	M 5, F 8	0.883^b^
Current smoking, *n* (%)	9 (33.3)	7 (50.0)	2 (15.4)	0.057^b^
Never smoking or quit smoking > 1 year ago, *n* (%)	18 (66.7)	7 (50.0)	11 (84.6)	
Diabetes type II, *n* (%)	3 (11.1)	3 (21.4)	0 (0)	0.077^b^
Cardiovascular disease, *n* (%)	11 (40.7)	4 (28.6)	7 (53.8)	0.182^b^
History of periodontitis^c^, *n* (%)	16 (59.3)	7 (50.0)	9 (69.2)	0.31^b^
Type of restoration, *n* (%)				0.354^b^
Single crown	9 (33.3)	6 (42.9)	3 (23.1)	
Implant supported fixed full denture	9 (33.3)	3 (21.4)	6 (46.2)	
Implant supported fixed partial denture	9 (33.3)	5 (35.7)	4 (30.8)	
Probing depth at deepest site, mm	7.4 (1.7)	6.9 (1.6)	8 (1.7)	0.084^a^
FMPS (%)	10.8 (13.0)	8.9 (9.0)	12.9 (16.5)	0.446^a^
FMBS (%)	3 (7.7)	1.1 (1.6)	5.1 (10.7)	0.18^a^
BoP (%)	11.9 (15.6)	12.2 (17.9)	11.5 (13.2)	0.913^a^
Implants per patients, *n*	4.5 (3.4)	4.1 (3.8)	4.9 (3.1)	0.564^a^
Teeth per patient, *n*	14.6 (10.3)	16.3 (11.1)	12.9 (9.4)	0.395^a^
Implants surgically treated for peri‐implantitis, *n*	40	21	19	0.92^a^
Implant surface characteristics, *n*				**0.023** ^b^ ^,^*
Non‐modified^d^	12	3	9	
Modified^e^	28	18	10	

*Note:* Values are presented as mean ± standard deviation unless otherwise stated. Significant difference are in bold letters between groups, *p* < 0.05.

^a^ Student's *t*‐test.

^b^ Chi‐square test.

^c^ History of periodontitis derived from patient files and radiographs and/or patient reported.

^d^ Turned (Nobel Biocare AB, Göteborg, Sweden).

^e^ TiUnite (Nobel Biocare AB); OsseoSpeed (Dentsply Sirona); SLA (Straumann Institute, Basel, Switzerland); Osseotite (Biomet 3i, Palm Beach, FL, USA).

### 3.2 Whole Genome Sequencing

Whole genome sequencing generated an average of 35.7 million (±8.2 million) counts.

**FIGURE 2:**
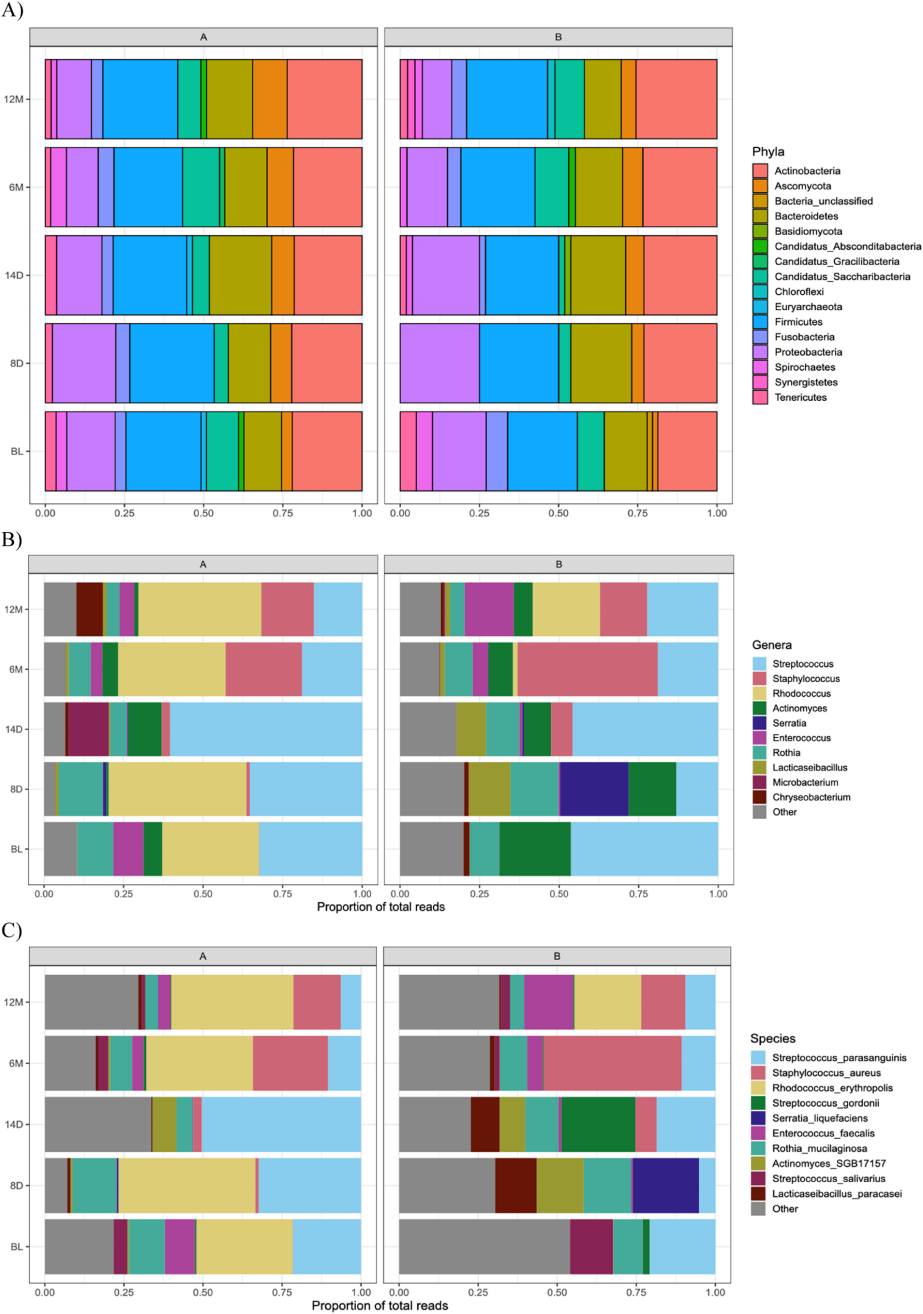
Bar charts of detected (A) phyla in proportion of samples, (B) genera read proportions and (C) species read proportions, in samples by time and intervention. All 16 detected phyla are shown in panel (A). The top 10 proportion of reads for each genus and species are show in panels (B) and (C). The rest of the genera and species not detected in the top 10 are shown as ‘Other’. A, amoxicillin and metronidazole; B, phenoxymethylpenicillin and metronidazole; BL, baseline; 8D, 8 days; 14D, 14 days; 6M, 6 months; 12M, 12 months.

These were classified into 16 phyla, 194 genera and 498 species. The overall distributions of the detected phyla and the 10 most present genera and species are shown in Figure 2A–C.

### 3.3 Alpha and Beta Diversity

Species alpha diversity (e.g., richness, evenness and Shannon diversity) of the salivary microbiome was investigated comparatively between the two interventions over time.

**FIGURE 3:**
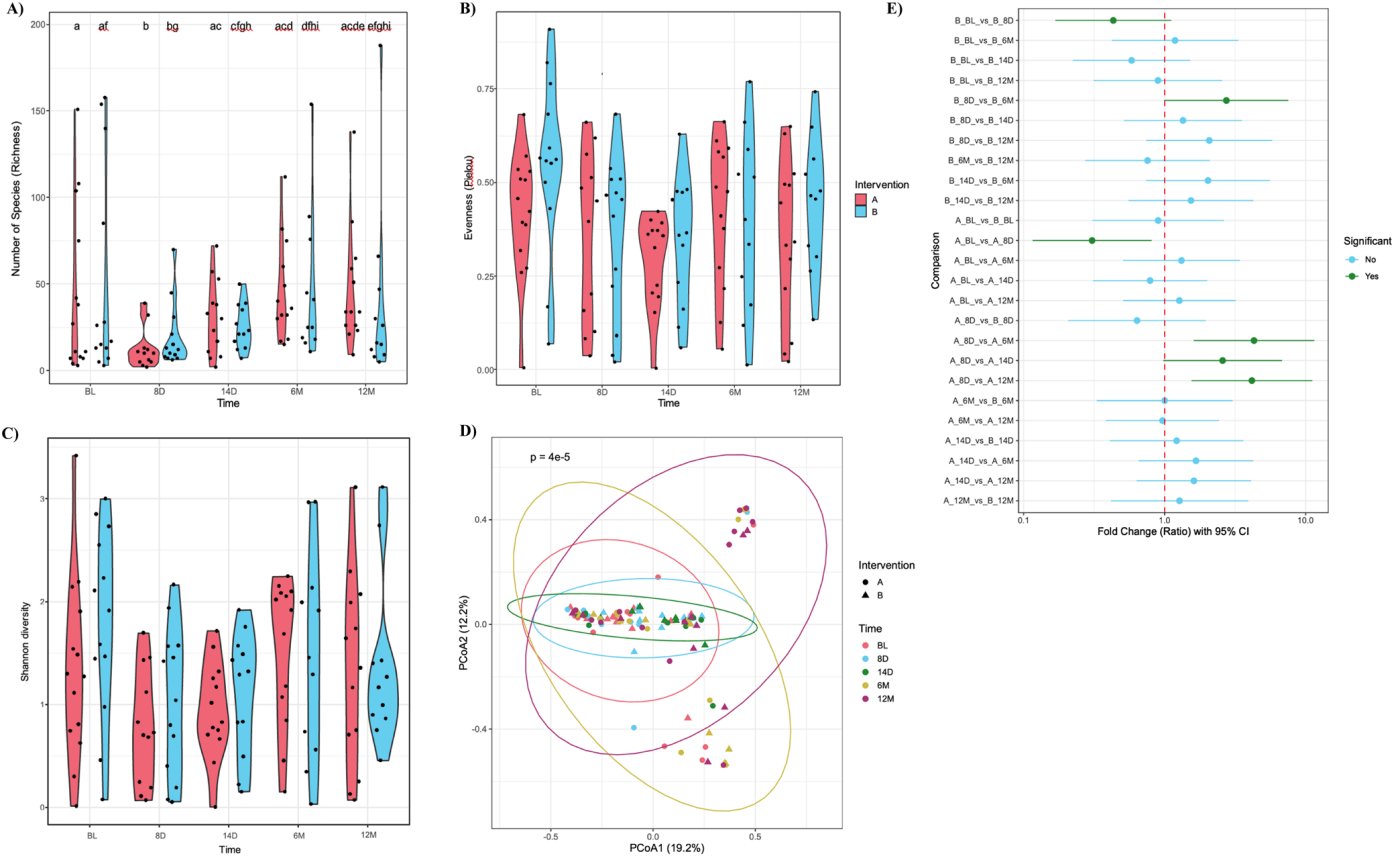
Alpha and beta diversity comparisons over time. Violin plots of the two interventions over time: (A) observed richness (ANOVA, *q* time = 0.00002; *q* time‐intervention = 0.557; *q* intervention = 0.762). Letters indicate comparisons with no statistically significant differences. (B) Pielou evenness (ANOVA, *q* = 0.100). (C) Shannon diversity (ANOVA, *q* = 0.04). (D) Principal coordinate analysis (PCoA) plot on Bray–Curtis distances (PERMANOVA, *q* = 0.00004) between the microbial community composition across samples (interventions and timepoints). Ellipses represent 95% CI. The distance between bullets/triangles reflects the magnitude of difference between the microbiota in samples. Intervention groups are distinguished by shape (bullet or triangle). (E) Richness pairwise comparisons on richness count data. Each dot in panels (A)–(C) represents one patient sample. Richness and pairwise comparisons performed with generaliged linear mixed model (GLMM) negative binomial, with post hoc FDR correction. Shannon and evenness were performed with linear mixed model (LMM). Intervention A, amoxicillin and metronidazole; B, phenoxymethylpenicillin and metronidazole.

Richness was statistically significantly reduced at 8 days relative to baseline for both groups. Richness increased by Day 14 compared to Day 8 (A, *q* < 0.05; B, *q* > 0.05). Both evenness and Shannon diversity exhibited comparable trends, and alpha diversity recovery was evident in both groups at 6 months (Figure 3A–C; Tables S1 and S2).

Pairwise comparisons of richness indicated significant within‐group changes across timepoints. However, no statistically significant differences were detected between interventions across timepoints (Figure 3E; Tables S1 and S2).

Regarding beta diversity, significant changes in microbial composition were observed across timepoints (*q* = 0.00004) but not between interventions (Figure 3D).

### 3.4 Changes in Differential Species Abundance Within Groups Between Timepoints

**FIGURE 4:**
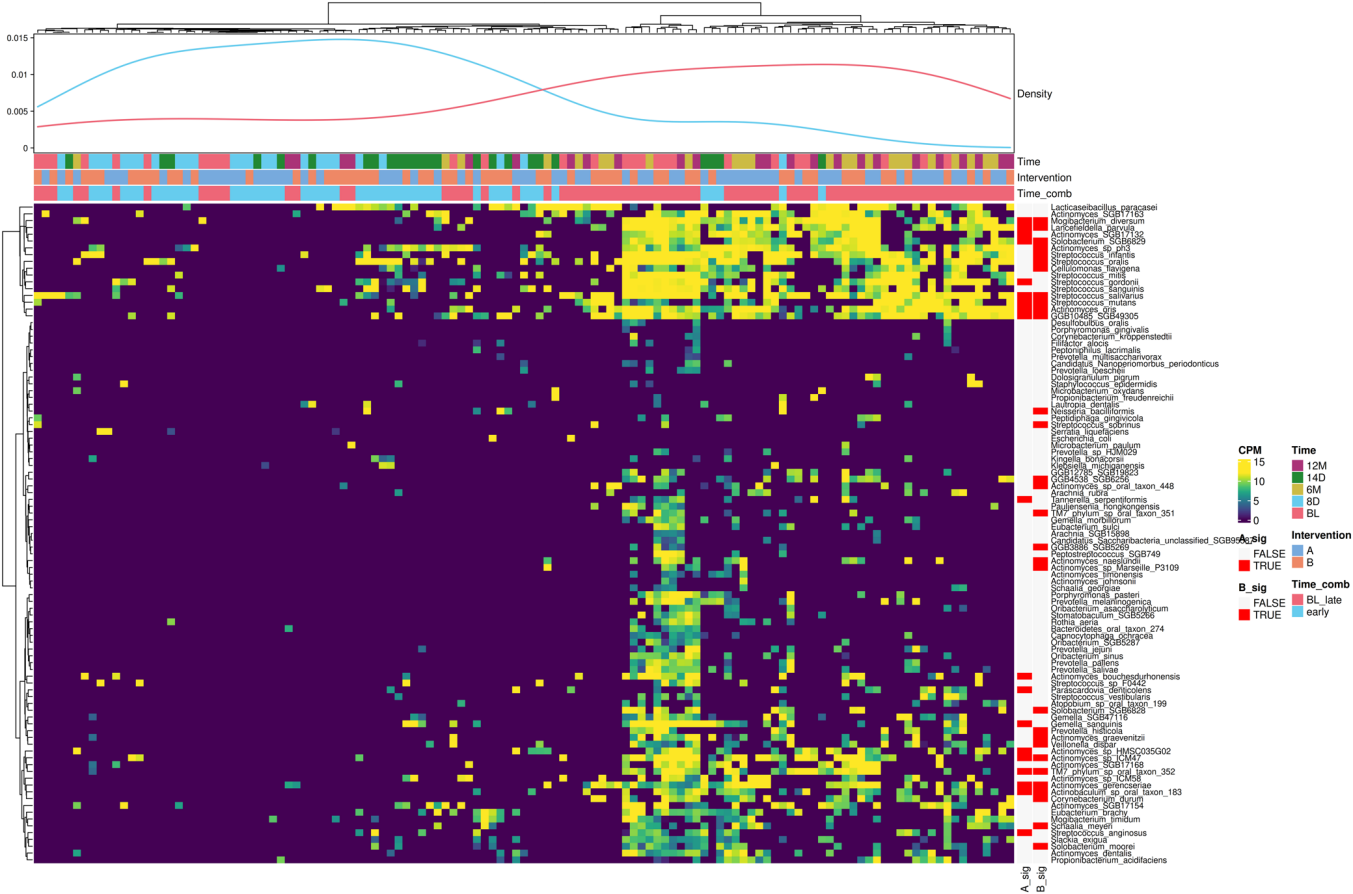
Heat map representing hierarchical clustering of species and samples based on normalized counts and standardised count values. Abundance ranges from low (dark purple) to high (yellow) for each significant species (row) and samples (column). Density analysis illustrates more similarity between species at baseline and late timepoints, for example, more clustering on the right‐hand side of the map under the red density line compared to early timepoints, low clustering on the left‐hand side of the map under the blue line. The bar chart in red shows significantly different species in Group A or Group B in any of the timepoints. Intervention, A, sig, amoxicillin and metronidazole, species significant in any of the timepoints are shown, *q* < 0.05. Intervention B, sig, phenoxymethylpenicillin and metronidazole, species significant in any of the timepoints are shown, *q* < 0.05. Time comb, time combinations; BL_late, baseline and late timepoints (6 months and 12 months); early (8 days and 14 days). Species were filtered to remove non/very low represented species. General linear model (GLM) was assessed for the species analysis and wardD2 (Euclidean distance method) for the hierarchical clustering. 8D, 8 days; 14D, 14 days; 6M, 6 months; 12M, 12 months; BL, baseline; CPM, normalized counts per million.

Significant changes in differential species abundance were observed for a total of 21 species in Group A and 37 species in Group B across timepoints. These changes were mainly observed between the shorter time intervals (baseline to Day 8, baseline to Day 14) compared to longer time periods (baseline to 6 months, baseline to 12 months). A total of 11 species were significant for both interventions (Figure 4). For detailed information see Table S3.

### 3.5 Changes in Differential Species Abundance Between Groups for Each Timepoint

A comparative analysis of the two interventions revealed a significantly lower abundance of 78 species in Group A compared to Group B at 8 days. Concurrently, 38 species exhibited higher abundance in Group A than in Group B. By Day 14, the trend shifted and demonstrated 80 species with higher abundance in Group A, while 50 species demonstrated lower abundance relative to Group B (Table S4). A total of seven species demonstrated significant shifts in abundance at three or more subsequent timepoints (Table 2).

**TABLE 2:** Significant changes in differential species abundance between Group A and Group B at ≥ 3 timepoints.

Group A versus Group B	8 days			14 days			6 months			12 months		
	Log FC	*p*	*q*	Log FC	*p*	*q*	Log FC	*p*	*q*	Log FC	*p*	*q*
Higher species abundance												
*Neisseria sicca*	7.605	0.000	**0.000**	8.215	0.000	**0.000**	7.295	0.000	**0.000**	0.358	0.765	0.929
*Actinomyces SGB17154*	3.575	0.005	**0.011**	3.971	0.003	**0.007**	3.532	0.008	**0.019**	−2.047	0.09	0.149
Lower species abundance												
*Streptococcus* sp.*F0442*	−18.554	0.000	**0.000**	−21.923	0.000	**0.000**	−3.318	0.008	**0.019**	1.871	0.132	0.212
*Capnocytophaga granulosa*	−17.552	0.000	**0.000**	−18.808	0.000	**0.000**	−14.564	0.000	**0.000**	1.163	0.337	0.465
*Actinomyces graevenitzii*	−10.886	0.000	**0.000**	−15.741	0.000	**0.000**	−3.541	0.005	**0.013**	0.071	0.952	1
*Alloprevotella tannerae*	−10.221	0.000	**0.000**	−7.393	0.000	**0.000**	−3.06	0.015	**0.032**	−4.597	0.002	**0.004**
*Lautropia dentalis*	−3.613	0.000	**0.000**	−6.810	0.000	**0.000**	−11.523	0.000	**0.000**	0	1	1

*Note:* Significant changes in differential species abundance between groups A and B on Day 8, Day 14, 6 months and 12 months. Significant difference are in bold letters between groups. *q* < 0.05. EdgeR analysis using general linear model (GLM) pipeline with trimmed mean of *M*‐values (TMM) was used as statistical method. Species were filtered to remove non/very low represented species.

Abbreviations: A, amoxicillin and metronidazole; B, phenoxymethylpenicillin and metronidazole; logFC, log_2_ fold‐change.

### 3.6 Treatment Success

No significant difference in clinical treatment success was observed between Group A and Group B (A: 83%, B: 69%, *p* = 0.645) when using the pre‐defined composite endpoints (Table 3).

**TABLE 3:** Treatment success at 12 months between antibiotic groups.

	Patient level, *n* = 25	
	Success	Failure
Group A	10 (83%)	2 (17%)
Group B	9 (69%)	4 (31%)
Total	19 (76%)	6 (24%)
*p*	0.645^a^	

*Note:* Treatment success is defined as patients presenting with no pocket depth > 5 mm, maximum one implant surface with bleeding on probing, no suppuration and no additional bone loss > 0.5 mm. Values are shown as number of patients (%) with complete radiological data. *p‐*value < 0.05 for statistical significance.

^a^ Fisher's exact test, two‐sided.

## 4 Discussion

This exploratory study was designed to descriptively investigate changes in the saliva microbiome following peri‐implant surgery with two beta‐lactam penicillin variants—one narrow‐spectrum and one broad‐spectrum—both combined with a nitroimidazole derivative.

WGS is highly sensitive, sequencing all DNA including that of low‐abundance, transient and non‐viable organisms and reagent contaminants, thereby detecting species missed by culture‐based methods. Noteworthy, *Serratia liquefaciens* , a non‐commensal linked to opportunistic infections, was found in high proportions but only in four different patients (Group A, *n* = 1 (Day 8); Group B, *n* = 2 (Day 8) and *n* = 1 (Day 14)). This detection of non‐commensal species could represent low‐level contamination or transient passage. However, its higher relative abundance in certain patients indicates the possibility of a genuine biological signal.

Species richness was directly affected in both groups within 8 days from the start of antibiotic treatment. A more marked decrease in evenness was observed for Group A at Day 14 compared to Group B, possibly demonstrating more species dominance in Group A and a more resilient microbial community in Group B. At 6 and 12 months, richness seemed to have recovered to the initial state in both groups.

Higher richness in healthy peri‐implant sites compared to diseased sites has commonly been described (Chun Giok and Menon 2023). This distinction was not evaluated in the present trial, but a significant proportion of the included patients achieved substantial clinical treatment success and hence progressed from a diseased state to a healthier state, which reflects both the mechanical and pharmacological disruption of the dysbiotic biofilm. Saliva is effective in a broad screening of the microbiome. However, its precision in reflecting specific site–related pathogens is limited (Bao et al. 2025). Our results align more with those of Belstrøm and coworkers, who reported a significant decrease in alpha‐diversity in subgingival and salivary microbiotas after periodontal treatment at 2 and 6 weeks and found that salivary levels of certain periopathogens can indicate subgingival colonisation (Belstrøm et al. 2018). In contrast, Yamanaka and coworkers reported that supragingival plaque microbiota has limited effect on the composition of salivary bacterial population after therapy (Yamanaka et al. 2012).

In the present trial, statistically significant differences were proven for several species between groups. *Alloprevotella tannerae*, which has been linked to periodontitis especially in the elderly (Schwartz et al. 2021), was the single observed species with lower abundance in Group A versus Group B over all timepoints. Additionally, *Streptococcus* sp. *F0442, Capnocytophaga granulosa, Actinomyces graevenitzi*, and *Lautropia dentalis—*all anaerobes or facultative anaerobes—had lower abundance from 8 days to 6 months.

Inversely, higher abundance of the aerobic *Actinomyces SGB17154* and microaerophilic *Neisseria sicca*, was noted up to 6 months. *Actinomyces* species plays emerging roles in many specific infections, but the clinical relevance of this particular strain is uncertain (Könönen 2024). *Neisseria sicca* is a commensal oropharyngeal species and considered a non‐pathogen although case reports have found an association with endocarditis (Cheng et al. 2024; Wang 2025). Additionally, members of the genus *Neisseria* are capable of horizontal gene transfer, which can result in the acquisition of antibiotic resistance (Manoharan‐Basil et al. 2022). The peak in species abundance between groups at 14 days could indicate either a healthy recovery of the normal microflora or an unhealthy imbalance (Liang et al. 2019). The explanation of the difference between groups may also be of technical detail, especially given the small sample size of the present cohort as well as individual differences, rather than the actual antibiotic effect on certain species.

Studies using 16S rDNA sequencing have shown that amoxicillin and metronidazole affect Fusobacteria, Saccharibacteria (TM7) and Bacteroidetes and cause variable changes in Actinobacteria, Firmicutes and Proteobacteria (Ferrer et al. 2017; Lazarevic et al. 2013; Menon et al. 2019; Zaura et al. 2015). Changes in typical periodontal pathogens like *Treponema* spp. and *Porphyromonas* spp. decrease while *Haemophilus* spp. and *Veionella* spp. increase during antibiotic treatment (Bizzarro et al. 2016; Hagenfeld et al. 2018). However, most anaerobic microbiota return to pre‐exposure levels within 2–4 weeks after antibiotics, with outcomes influenced by the antibiotics' properties, dose, administration route and activity (Rashid et al. 2013; Sullivan et al. 2001).

The major limitations of this study are the relatively small sample size, as well as the absence of a placebo group. These may weaken the study conclusions on detailed species‐level microbiological differences between the two interventions, but they are less likely to affect the outcome of the overall community composition, such as diversity measures. Furthermore, the post‐operative use of chlorhexidine (CHX) alongside antibiotics may have increased the impact on alpha diversity, complicating the interpretation of isolated effects of antibiotics, surgery or chlorhexidine on the saliva microbiome. CHX is considered the gold standard antiseptic in dentistry and oral care (Garrido et al. 2023; Jones 1997). Studies show that extended use of CHX lowers the abundance of multiple oral bacteria, such as *Actinomyces* spp. (do Amaral et al. 2023) and *Prevotella* spp. (Bescos et al. 2020). Bartsch and co‐workers found decreased alpha diversity and increased tetracycline resistance genes after using 0.2% chlorhexidine mouthwash twice daily for 4 weeks post periodontal surgery (Bartsch et al. 2025). CHX also increased *Streptococcus* spp. levels in in vivo studies (Bartsch et al. 2024). Despite the equal CHX exposure in both groups, changes between days 8 and 14 suggest antibiotic effects remain consistent. Additionally, CHX is expected to have short‐term localised effects, while antibiotics are typically systemic. Metronidazole, in particular, maintains above‐minimum inhibitory concentration for extended periods (Reissier et al. 2023). Variables such as age, gender and diet may additionally influence the results (Wells et al. 2022). Furthermore, host factors such as enzyme expression, saliva flow, small molecules and taste receptor differences, all influence the oral microbiota composition (Davenport 2017).

The efficacy of using metronidazole as a single adjunctive agent limited to specific peri‐implant cases is supported by the EFP S3 level clinical practice guidelines (Herrera et al. 2023).

Although this study is descriptive and does not propose specific antibiotic regimens, it hints that narrow‐spectrum antibiotics could be worth considering from an antimicrobial stewardship perspective.

## 5 Conclusion

The composition and structure of the salivary microbiome appeared to be similarly affected by both systemic antibiotic regimens used adjunctively to peri‐implant surgery. However, given the descriptive nature of this study and the limited sample size, it would be premature to draw firm distinctions between the regimens. Given these considerations, it may be helpful for clinicians to reflect on the potential risks and anticipated benefits of antibiotic use when treatment planning. Further research is valuable to deepen our understanding of the potential long‐term effects that combined antibiotic regimens may have on the salivary resistome.

## Author Contributions

Caroline Riben Grundström contributed to the design/study planning, data acquisition, data analysis and interpretation, funding acquisition and drafting and revising the manuscript. Bodil Lund was involved in conception and design of the study, ethical/legislator permit, setting up of study site, data analysis, funding acquisition, supervision and drafting and critically revising the manuscript. Nagihan Bostanci was involved in design/study planning, data analysis and interpretation, supervision and drafting and critically revising the manuscript. Angelika Silbereisen was involved in sample handling and preparation and drafting and revising the manuscript. Alexandra Pennhag was responsible for the DNA extraction and revising the manuscript. Yinghua Zha was responsible for planning and sequencing of DNA samples and revising the manuscript. Ina Schuppe Koistinen was responsible for the microbiome analysis and revising the manuscript. Anastasios Damdimopoulos conducted bioinformatic and biostatistical analyses and contributed to the revision of the manuscript. Georgios N. Belibasakis was involved in the design/study planning, data analysis and interpretation, supervision and drafting and critically revising the manuscript. Margareta Hultin contributed to the conception and design of the study, ethical/legislator permit, setting up of study site, data analysis, project administration including web publication and external monitoring, funding acquisition, supervision and drafting and critically revising the manuscript. All authors gave final approval and agreed to be accountable for all aspects of the work.

## Funding

This work was supported by Folktandvården Region Uppsala and grants from Region Uppsala, the Swedish Dental Association, the Public Health Agency of Sweden, Steering Group for Collaborative Odontological Research at Karolinska Institutet and Stockholm City County, and the Centre for Innovative Medicine (CIMED).

## Ethics Statement

The study was conducted in accordance with all relevant points of the Declaration of Helsinki of the World Medical Association.

## Conflicts of Interest

The authors declare no conflicts of interest.

## Supporting information


**Data S1:** Materials and methods.

**Figure S1:** Descriptive details of saliva sample collection for all included patients over time. Each bar represents one patient with samples in Group A or Group B. Each patient was sampled at the following timepoints: baseline, Day 8, Day 14, 6 months and 12 months.

**Table S1:** Alpha diversity summary statistics for groups A and B over time.

**Table S2:** Richness pairwise analyses within and between groups A and B over time.

**Table S3:** Significant changes in differential species abundance within Group A and Group B compared to baseline.

**Table S4:** Significant changes in differential species abundance between Group A and Group B over time.

## Data Availability

The data that support the findings of this study are available from the corresponding author upon reasonable request.
