# Supplementary material for: Systemic Antibiotics in the Surgical Treatment of Peri‐Implantitis: Impact on the Salivary Microbiome
Source: J Clin Periodontol. 2026 May 25;53(8):1265–77. doi: 10.1111/jcpe.70143 (PMC13371835; doi:10.1111/jcpe.70143)
Supplement: Supplementary file 1 — Data S1: Materials and methods. [file JCPE-53-1265-s003.docx]

**Supporting information (S1)**

**TITLE**

**Systemic antibiotics in the surgical treatment of peri-implantitis: Impact on the salivary microbiome**

**AUTHORS**

*Caroline Riben Grundström, Bodil Lund, Nagihan Bostanci, Angelika Silbereisen, Alexandra Pennhag, Ina Schuppe Koistinen, Yinghua Zha, Anastasios Damdimopoulos, Georgios N. Belibasakis, Margareta Hultin.*

**S1 Materials and methods**

**S1.1 Inclusion and exclusion criteria**

Included patients were ≥18 years of age with peri-implantitis (defined as PPD ≥ 6 mm, with BOP and/or SOP and marginal bone level change ≥2 mm on at least one implant in function ≥1 year), full-mouth plaque score ≤30%, with healthy or treated periodontal conditions, enrolled in a regular supportive program. Exclusion criteria were as follows:

- Known allergy to penicillins
- Antibiotic drug interactions
- Use of antibiotics during last 3 months
- Pregnancy or breastfeeding
- Immunosuppression
- Diabetics with poor glycemic control (HbA1c>75 mmol/mol)
- Intravenous bisphosphonate treatment
- Nervous system diseases
- Alcohol abuse
- Lactose/galactose intolerance
- Medical conditions/medications which, according to the examiner, could interfere with the outcome of the study or affect patient safety
- Untreated periodontal conditions
- Incapability of performing oral hygiene due to physical or mental disorders
- Implants with bone loss exceeding two-thirds of the implant length
- Implant mobility
- Xerostomia
- Slow bowel motion

**S1.2 Sample size**

**S1.3 Clinical saliva sample collection and processing**

After the saliva samples had been delivered to the dental clinic they were transported to a

-80°C freezer at the core facility, ANA Futura, Karolinska Institute, Huddinge for long-term storage.

**S1.4** **DNA extraction**

The samples were thawed and 300 $\mu L$ DNA/RNA shield was added. 800 $\mu L$was then transferred to a ZR BashingBead lysis tube (0,1 & 0,5 mm) (Zymo Research). A positive control of 75 µl of ZymoBIOMICS Community standard (Zymo Research) in 725 $\mu L$DNA/RNA shield (Zymo Research) and a negative control of 800 µl of DNA/RNA shield were included.   All samples were bead beat in a FastPrep24 5G, 5 times at 6,0 m/sec for 1 min with 5 min of pause between beatings resulting in a total bead beating time of 5 min. Thereafter, the samples were centrifuged at 10 000 g for 1 min and 200 $\mu L$of the sample were added to a deep well plate and purified in a Tecan Fluent according to ZymoBIOMICS 96 MagBead DNA protocol (Zymo Research).

**S1.5 Library preparation**

Due to the low microbial biomass in saliva DNA samples, the thermo-acidophilic bacterium *Alicyclobacillus acidiphilus,* was added to samples with a lower than minimum detected DNA concentration, to aid in the detection of low amount of microbial DNA. For samples with concentrations meeting the required 50ng DNA input, 0.5ng of *A. acidiphilus* that equals 1% of the total DNA input, was added. Equal amounts of DNA from each sample were pooled together, followed by circulating 1pmol of these pooled libraries using MGI Easy Circularization kit (MGI Tech, China), and subjected to 150bp paired-end sequencing on the DNBSEQ G400 sequencing instrument (MGI Tech, China) according to manufacturer's instructions.

**S1.6 Statistical analyses**

Counts and abundance data generated from MetaPhlAn4 were imported into R Statistical Software (version 4.4.2; R Core Team 2024) and Bioconductor (version 3.21) for downstream analysis. For differential species analyses across timepoints which requires absolute abundance estimates, relative abundances were converted to estimated read counts by multiplying each species’ relative abundance by the corresponding sample’s total number of non‑host sequencing reads. This approach accounts for variation in sequencing depth and provides a standardized basis for assessing temporal changes in microbial composition.

Relative abundance alone loses information about sequencing depth. Two samples might have the same percentages but very different reads. By converting percentages to estimated counts the analysis can account for sequencing depth differences and the statistical method (edgeR) can be used.

edgeR is specifically designed to work with **count data** from sequencing experiments.

Permutational Multivariate Analysis of Variance (PERMANOVA) with adonis2 function, all from the vegan package, with 99999 permutations used.
